# The forkhead box transcription factor FoxP4 regulates thermogenic programs in adipocytes

**DOI:** 10.1016/j.jlr.2021.100102

**Published:** 2021-08-09

**Authors:** Luce Perie, Narendra Verma, Elisabetta Mueller

**Affiliations:** Division of Endocrinology, Diabetes and Metabolism, Department of Medicine, New York University Grossman School of Medicine, New York, NY, USA

**Keywords:** FoxP4, UCP1, brown fat, beige fat, thermogenesis, β-adrenergic stimuli, HSF1, heat shock element, chromatin immunoprecipitation, luciferase assays, ZNF638, BAT, brown adipose tissue, ChIP, chromatin immunoprecipitation, eWAT, epididymal white adipose tissue, FoxP4, Forkhead box P4, HEK-293, human embryonic kidney-293, HSE, heat shock element, HSF1, heat shock factor protein 1, IBMX, isobutylmethylxanthine, scWAT, subcutaneous white adipose tissue, siLuc, siRNA luciferase, SVF, stromal vascular fraction, TBST 0.1% buffer, 50 mM Tris-HCl, 150 mM NaCl, pH 7.4%, and 0.1% Tween-20, UCP1, uncoupling protein 1

## Abstract

Forkhead box transcription factors have been shown to be involved in various developmental and differentiation processes. In particular, members of the FoxP family have been previously characterized in depth for their participation in the regulation of lung and neuronal cell differentiation and T-cell development and function; however, their role in adipocyte functionality has not yet been investigated. Here, we report for the first time that Forkhead box P4 (FoxP4) is expressed at high levels in subcutaneous fat depots and mature thermogenic adipocytes. Through molecular and gene expression analyses, we revealed that FoxP4 is induced in response to thermogenic stimuli, both in vivo and in isolated cells, and is regulated directly by the heat shock factor protein 1 through a heat shock response element identified in the proximal promoter region of FoxP4. Further detailed analysis involving chromatin immunoprecipitation and luciferase assays demonstrated that FoxP4 directly controls the levels of uncoupling protein 1, a key regulator of thermogenesis that uncouples fatty acid oxidation from ATP production. In addition, through our gain-of-function and loss-of-function studies, we showed that FoxP4 regulates the expression of a number of classic brown and beige fat genes and affects oxygen consumption in isolated adipocytes. Overall, our data demonstrate for the first time the novel role of FoxP4 in the regulation of thermogenic adipocyte functionality.

Excessive adipose tissue expansion leads to obesity and is associated with increased risk to develop a number of metabolic disorders, including type 2 diabetes, hypertension, and cardiovascular disease (1). It has been now established that three different types of adipocytes—white, beige, and brown—constitute fat tissues. These cells have been shown to be characterized by specific gene signatures and distinct functions and to be involved in the regulation of metabolic processes, including energy storage, glucose uptake, and thermogenesis (2, 3). Specifically, brown adipocytes present in brown fat (brown adipose tissue [BAT]) located in the interscapular region in mice and beige fat cells present in subcutaneous fat (subcutaneous white adipose tissue [scWAT]) have been shown to contribute to energy expenditure and play crucial roles in the regulation of energy homeostasis (4), whereas white adipocytes present in the visceral fat depot (epididymal white adipose tissue [eWAT]) are involved in energy storage (2, 3, 4, 5, 6). Given the evidence that we and others have provided to demonstrate that brown/beige adipocytes are present in adult humans and that they play a critical role in energy and glucose homeostasis (5, 7, 8, 9, 10), thermogenic fat cells have been now considered a possible target for the treatment of metabolic dysfunction.

Forkhead box factors belong to a large family of transcription factors comprising more than 50 members characterized by a homologous DNA-binding domain. They have been involved in development and differentiation and, specifically, members of the Foxo and Foxa family have been shown to participate in metabolism. In particular, transcription factors such as Foxo1 were found to modulate uncoupling protein 1 (UCP1) expression (11) in adipocytes and regulate insulin signaling and metabolism (11, 12, 13, 14). In addition, work performed in our laboratory has demonstrated that Foxa3 promotes adipocyte differentiation in vitro and that its ablation decreases adiposity in aging mice and extends their life span (15, 16).

The members of the FoxP subfamily, which includes four members—FoxP numbered 1 through 4, contain a conserved leucine zipper domain and a zinc finger motif, in addition to the characteristic winged helix/forkhead DNA-binding module (17) that gives the name to the entire Fox family. Each of these FoxP factors have been shown to be expressed in select organs and tissues with some degree of overlap and involved in the regulation of diverse biological processes such as pulmonary functionality and immunity (18, 19, 20, 21, 22). Specifically, FoxP1, FoxP2, and Forkhead box P4 (FoxP4) have been known to be involved in developmental programs in the lung and brain (19, 23, 24, 25), whereas FoxP3 has been shown to be predominantly expressed in T cells and contribute to their differentiation (20, 26). Despite this knowledge, to date, there is paucity of information on the levels of expression of FoxP family member in adipose tissues and on their effects on fat cell functionality in general, and, specifically, on thermogenesis. Here, we show for the first time that the forkhead box factor FoxP4 is expressed in thermogenic cells and that its levels are regulated by thermogenic cues via the transcription factor heat shock factor protein 1 (HSF1). Furthermore, our data provide for the first time evidence that FoxP4 is a novel regulator of thermogenic fat gene expression and function. Altogether, our studies demonstrate a critical role of FoxP4 in the regulation of adipocytes biology not previously appreciated.

## Materials and Methods

### Cell culture

10T1/2 and human embryonic kidney-293 (HEK-293) cells were obtained from American Type Culture Collection. Stromal vascular fraction (SVF) cells were isolated from scWAT adipose tissues of male mice and maintained in growth medium using DMEM (Corning; #10-013-CV), supplemented with 10% (v/v) FBS (Thermo Fisher Scientific; #NC0959573) and with 1% penicillin/streptomycin (Thermo Fisher Scientific; #15070063). Isolation of mouse SVF cells was performed as previously described (27). Briefly, dissected scWAT fat pads obtained from 9-week-old C57Bl/J6 male mice were washed in PBS and digested for 1 h in a solution containing 1 mg/ml of collagenase type IV (Roche; #10269638001); the resulting cells were filtered through a 70-μm cell strainer (BD Falcon; #352350) to eliminate clumps and centrifuged for 10 min at 200 *g*. The pelleted cells representing the SVF were resuspended in culture medium. To induce brown-like fat differentiation, confluent 10T1/2 cells and SVF cells were treated with induction medium containing DMEM supplemented with 10% FBS, 1% penicillin/streptomycin, 20 nM insulin (Sigma; #I1507), 1 nM T3 (Sigma; #T2877), 125 μM indomethacin (Sigma; #I7378), 1 μM dexamethasone (Sigma; #D4902), 1 μM rosiglitazone (Sigma; #557366-M), and 0.5 μM isobutylmethylxanthine (IBMX; Sigma; #I5879). The culture medium was replaced with maintenance medium containing DMEM supplemented with 10% FBS, 1% penicillin/streptomycin, 1 nM T3, and 20 nM insulin after 2 days for 10T1/2 cells or after 4 days for mouse SVF cells. We subsequently replaced the maintenance medium every 2 days, until cells were fully differentiated. To test the role of cAMP modulators in the regulation of FoxP4 levels, we treated brown fat differentiated cells with either vehicle or with 5, 10, or 20 μM of forskolin (Sigma; #F6886), 5, 10, or 20 μM of isoproterenol (Sigma; #I6504) or 125, 250, or 500 μM of IBMX, for 4 h before cells were harvested for molecular analysis or with doses and times otherwise noted in the legends. To obtain mature adipocytes, we dissected ScWAT fat pads of 9-week-old C57Bl/J6 male mice and washed in PBS containing 1% antibiotic and digested for 45 min in a solution containing 1 mg/ml of collagenase type IV (Roche; #10269638001); the resulting cells were filtered through a 100-μm cell strainer (BD Falcon; #352350) to eliminate clumps and centrifuged for 10 min at 200 *g*. Middle layer containing mature adipocytes and the pelleted cells representing the SVF were resuspended in lysis buffer for RNA extraction (Qiagen; catalog no. 74104).

### Mice

Male and female C57Bl/J6 mice (Jackson Laboratories; catalog no. #000664) were housed at 24°C in standard cages and fed standard chow diet, exposed to a 12-h light/12-h dark cycle and had free access to food and water. For cold exposure experiments, 9-week-old male and female mice were either kept at room temperature or at 4°C, with full access to fresh water, for up to 6 h. Mice were euthanized at the end of the cold exposure, and fat adipose tissues were collected for mRNA and protein analysis. For studies of the effects of β-adrenergic stimulation, 100 μl of either CL316,243 dissolved in saline, at the concentration of 1 mg/kg of body weight (Sigma; #C5976), or saline alone, were injected in 9-week-old mice intraperitoneally. Fat depots were collected 3 h after the injections. To study the effect of HSF1 gain of function in vivo on the levels of FoxP4, we injected adenoviruses expressing either control (CMV-GFP) or mouse HSF1 (HSF1-CMV-GFP) (generated by Vector Biolabs) as previously described (16). Briefly, a total of 50 μl of 5 × 10^9^ Pfu of each adenovirus diluted in saline were injected subcutaneously bilaterally into the scWAT fat pads of 9-week-old male mice, twice a week, for 2 weeks. Mice were sacrificed at the fourth day after the final viral delivery. The Institutional Animal Care and Use Committee at New York University Langone Medical Center approved the mouse experimental protocol for this study.

### Plasmids

The expression plasmid for FoxP4 was obtained from Origene (MR 221681) and the HSF1 plasmid from Addgene (#71724); the pGL3 luciferase reporter vector (#E1751) and the pRL *Renilla* luciferase control reporter vector (#E2231) were purchased from Promega. The UCP1 (2.1 kb) luciferase plasmid was a kind gift of Dr Evan Rosen (Beth Israel Deaconess Medical Center, Boston, MA) and Dr Sona Kang (U.C. Berkley).

### Transient transfections

For gene expression studies, we transfected differentiated 10T1/2 cells using DharmaFECT transfection reagent (Dharmacon; #T-2002-03), according to the manufacturer's instructions. Specifically, for gain-of-function experiments, brown-like differentiated 10T1/2 cells were transfected with 5 μg of either vector, HSF1, or FoxP4 expression plasmids and harvested 2 days later. For knockdown experiments, brown-like differentiated 10T1/2 cells were transfected with 35 nM of siRNA luciferase (siLuc; Dharmacon; #D002050-01-20), siRNA HSF1, or siFoxP4 and harvested 3 days later. To test the effect of FoxP4 on brown fat differentiation, undifferentiated 10T1/2 cells were transfected with 35 nM of siLuc or siFoxP4. About 24 hours after transfection, cells were induced to differentiate and harvested 5 days later.

### RNA isolation and RT-PCR analysis

Extraction of total RNA from cultured cells and fat tissues was achieved using RNeasy Midi Kit (Qiagen; #75144) and TRIzol (Thermo Fisher Scientific; #15596018), respectively. About 1 μg of total RNA was reverse transcribed using the iScript™ cDNA Synthesis Kit (BioRad; #1708890). For gene expression analysis, we performed real-time quantitative PCR in triplicate using 25 ng of complementary DNA and 300 nM of primers with the iQ™ SYBR® Green Supermix (BioRad; #1708880), according to manufacturer's protocols. The relative mRNA quantification was calculated via the ΔΔCt method with normalization of each sample to the average change in cycle threshold value of the control 36B4 gene. We used the following primers for quantitative PCR analysis: FoxP4—Forward: CGACATGATGGTGGAATCTG, Reverse: TGTTTGCTGTCATTGTTCCC; HSF1—Forward: AGGCAGGAGCATAGATGAGA, Reverse: AGGATGGAGTCAATGAAGGC; Ucp1—Forward: GGCCCTTGTAAACAACAAAATAC, Reverse: GGCAACAAGAGCTGACAGTAAAT; 36B4—Forward: GCTTCATTGTGGGAGCAGAC, Reverse: ATGGTGTTCTTGCCCATCAG; PGC1α—Forward: ACCATGACTACTGTCAGTCACTC, Reverse: GTCAC AGGAGGCATCTTTGAAG; PRDM16—Forward: CCACCAGCGAGGACTTCAC, Reverse: GGAGGACTCTCGTAGCTCGAA; Cidea—Forward: TGACATTCATGGGATTGCAGAC, Reverse: CGAGCTGGATGTATGAGGGG; CytC—Forward: AAATCTCCACGGTCTGTTCGG, Reverse: GGGTATCCTCTCCCCAGGTG; Mcad—Forward: ATGACGGAGCAGCCAATGAT, Reverse: TCGTCACCCTTCTTCTCTGCTT; Cpt1α—Forward: TTGCCCTACAGCTCTGGCATTTCC, Reverse: GCACCCAGATGATTGGGATACTGT; Cox4β—Forward: CTGCCCGGAG TCTGGTAATG, Reverse: CAGTCAACGTAGGGGGTCATC; Elovl3—Forward: TTCTCACGCGGGTTAAAAATGG, Reverse: TCTCGAAGTCATAGGGTTGCAT; ZNF638—Forward: ATTGAGAGCTGTCGGCAGTTA, Reverse: GGAATGAGAACGT CTTCTTGGAG; PPARα—Forward: TCGAGGAAGGCACTACACCT, Reverse: TCTTCCCAAAGCTCCTTCAA; aP2—Forward: ACACCGAGATTTCCTTCAAACTG, Reverse: CCATCTAGGGTTATGATGCTCTTC; C/EBPα—Forward: GAACAGCAACGAGTACCGGGTA, Reverse: GCCATGGCCTTGACCAAGGAG; and PPARγ—Forward: AGTCTGCTGATCTGCGAGCC, Reverse: CTTTCCTGTCAAGATCGCCC.

### Western blot analysis

Whole cell extracts were obtained from cultured cells and tissues with RIPA buffer (20 mM Tris, 150 mM NaCl, and 1% NP-40), supplemented with a cocktail of protease inhibitors (Thermo Fisher Scientific; #PIA32953). About 20 μg of protein lysates were run on a 10% SDS-polyacrylamide gels and transferred onto 0.45 μm PVDF membranes (Millipore; #IPVH00010). Membranes were blocked using a solution containing 5% nonfat dry milk (w/v) resuspended in TBST 0.1% buffer (50 mM Tris-HCl, 150 mM NaCl, pH 7.4%, and 0.1% Tween-20) for 1 h at room temperature and were subsequently incubated with primary antibodies at 4°C overnight in a solution containing 1% BSA resuspended in TBST 0.1% buffer (Thermo Fisher Scientific; #BP9703-100). We used the following antibodies at the dilutions indicated: anti-FoxP4 (Proteintech; #16772-1-AP) at 1:1,000; anti-HSF1 (CST; #4356) at 1:1,000; anti-UCP1 (Abcam; #ab10983) at 1:1,000, and antivinculin (Proteintech; #66305-1-Ig) at 1:2,000. The membranes were washed three times in TBST 0.1% (v/v) after incubation with the primary antibody and subsequently incubated at room temperature for 1 h with a 1:20,000 dilution of antirabbit (#1706515) or antimouse (#1706516) IgG horseradish peroxidase-conjugated (BioRad) in TBST 0.1% containing 2% nonfat dry milk (w/v). Immunoblots were developed after four additional washes in TBST 0.1% (v/v), using an enhanced chemiluminescence kit (GE Healthcare; #RPN2108) on Hyblot CL autoradiography films (Thomas Scientific; #E3012), using an X-ray film developer (Konica Minolta; #SRX-101A).

### Chromatin immunoprecipitation assays

Chromatin immunoprecipitation (ChIP) analysis of 10T1/2 cells differentiated into brown-like adipocytes was performed according to the manufacturer's protocol (Millipore; #17-295), as previously described (27). Briefly, differentiated cells were treated with 1% formaldehyde to crosslink protein to DNA. Cells were then washed with ice-cold PBS buffer containing protease inhibitors (Millipore; #20-283), and subsequently harvested and sonicated for 30 min using a Diagenode Bioruptor®. The protein-DNA complexes were immunoprecipitated at 4^o^C overnight using an anti-HSF1 antibody or an anti-FoxP4 antibody or with an anti-IgG control antibody (Millipore; #17-295). After the cross-linked protein–DNA complexes were incubated with protein A agarose beads (Millipore; #16-201C), they were eluted for 15 min at room temperature using Elution Reagent C (Millipore; #20-294). DNA fragments were purified and amplified by RT-PCR using the primers listed later, designed specifically to recognize the area spanning the putative heat shock element (HSE) identified in the proximal region of the FoxP4 promoter, in position -240 to -233, and the putative forkhead response element identified in the proximal UCP1 promoter in position -479 to -472: PromFoxP4—Forward: CTTCAACAGCCTTGGACCAG, Reverse: CTTCAACAGCCTTGGACCAG; PromUCP1—Forward: CTTCAACAGCCTTGGACCAG, Reverse: CTTCAACAGCCTTGGACCAG; and Actb—Forward: TGAGGTACTAGCCACGAGAGAG, Reverse: ACACCCGCCACCAGGTAAGCA.

### Luciferase assay

For luciferase assays, we plated 20,000 HEK-293 cells per well of a 12-well tissue culture plate, and 24 h later, cells were cotransfected with 0.5 μg of the UCP1 luciferase reporter plasmid and with vector expression plasmid or FoxP4 (0.5 μg) and 50 ng of Renilla reporter plasmid, using Lipofectamine 2000 (Thermo Fisher; #11668019). About 48 h after transfection, cells were harvested for luciferase assays. The luciferase reporter activity was measured using the Dual-Luciferase Reporter Assay System Kit (Promega; #E1910). Luciferase activity was detected using the SpectraMax 96 well Plate Reader (Molecular Devices, CA), and the values were normalized to Renilla activity.

### Cellular metabolic rates

Cellular metabolic rates were measured using an XFe24 Analyzer (Seahorse Agilent). For FoxP4 overexpression studies, differentiated 10T1/2 cells were transfected with either vector or FoxP4 expression plasmid, whereas for knockdown studies, either control siRNA (siLuc) or siFoxP4 were transfected in these cells. Respiration was measured under basal conditions, following the addition of ATP synthase inhibitor oligomycin, the mitochondrial uncoupler carbonyl cyanide-4-(trifluoromethoxy)-phenylhydrazone, or the complex III inhibitor antimycin A/rotenone, as per vendor's protocol (Agilent; catalog no. 103015-100).

### Statistical analysis

All the results were expressed as mean ± SE, unless otherwise noted. Different groups were compared via Student's *t*-test or by one-way analysis of variance. *P* values <0.05 were considered statistically significant. All statistical analyses were performed using Prism 7 software (GraphPad Software, Inc).

## Results

### FoxP4 is expressed in fat tissues and isolated adipocytes

**Fig. 1:**
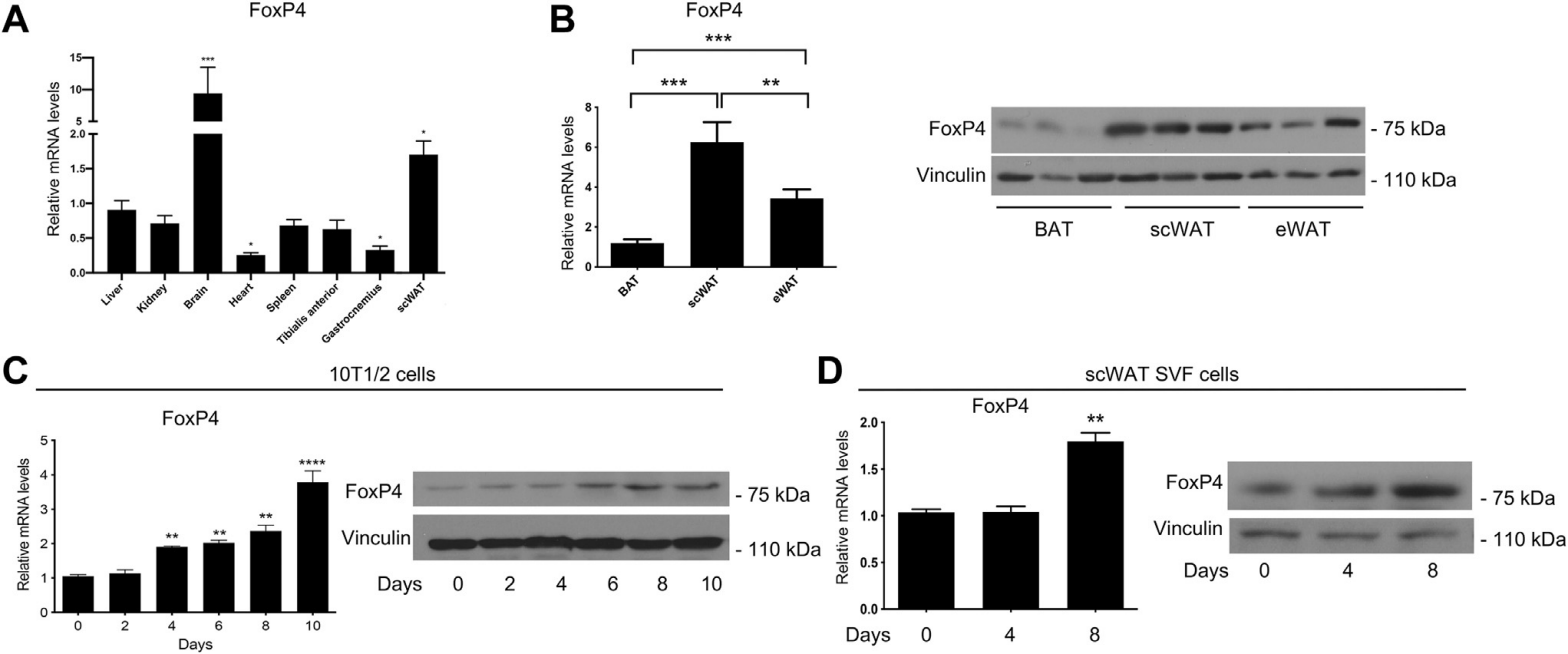
FoxP4 is expressed in thermogenic tissues and fully differentiated brown-like adipocytes. A: Relative FoxP4 mRNA levels in tissues of 9-week-old male mice fed a normal diet (n = 4). Vinculin was used as a loading control. B: Relative FoxP4 RNA and protein levels in BAT, scWAT, and eWAT fat depots. Vinculin was used as a loading control. C and D: Relative mRNA and protein levels of FoxP4 during a time course of brown differentiation of 10T1/2 cells (C) and SVF cells isolated from scWAT (D). Vinculin was used as a loading control. Results are expressed as a mean ± SEM from three independent experiments. ∗*P* < 0.05∗*P* < 0.005; and ∗∗∗*P* < 0.001. BAT, brown adipose tissue; eWAT, epididymal white adipose tissue; FoxP4, Forkhead box P4; scWAT, subcutaneous white adipose tissue; SVF, stromal vascular fraction.

FoxP4 has been previously shown to be expressed, and to exert its function, in neurons, intestine, and immune cells (18, 21, 22), but whether FoxP4 is expressed in fat depots and if it plays a role in the differentiation and function of adipocytes is currently unknown. To assess FoxP4 tissue distribution and compare its levels in different organs, we initially performed gene expression analysis to detect FoxP4 in 9-week-old adult male mice fed a normal diet. As shown in Fig. 1A, we detected elevated amounts of mRNA of FoxP4 specifically in scWAT, compared with other fat depots, and other metabolic organs. Analysis of FoxP4 in the brain performed in parallel served as a positive control, given that FoxP4 levels in this organ are known to be the highest based on previous reports (28). RNA and Western blot analysis of the three fat depots confirmed that FoxP4 is present at higher levels in scWAT, compared with eWAT and BAT (Fig. 1B). To assess whether the expression of FoxP4 is regulated during brown-like adipocyte differentiation, we performed time course studies of differentiating 10T1/2 cells. The analysis of RNA and protein levels of FoxP4 at different time points during adipogenesis revealed that FoxP4 levels are at the highest after differentiation is achieved, whereas FoxP4 expression appears low in undifferentiated cells (Fig. 1C). Similar results were obtained in differentiating SVF cells derived from scWAT (Fig. 1D). Further analysis of scWAT revealed that FoxP4 levels are higher in mature adipocytes compared with SVF cells (Fig. S1). These data demonstrate that FoxP4 is expressed in fat tissues and fully differentiated thermogenic adipocytes.

### FoxP4 levels are regulated by thermogenic stimuli

**Fig. 2:**
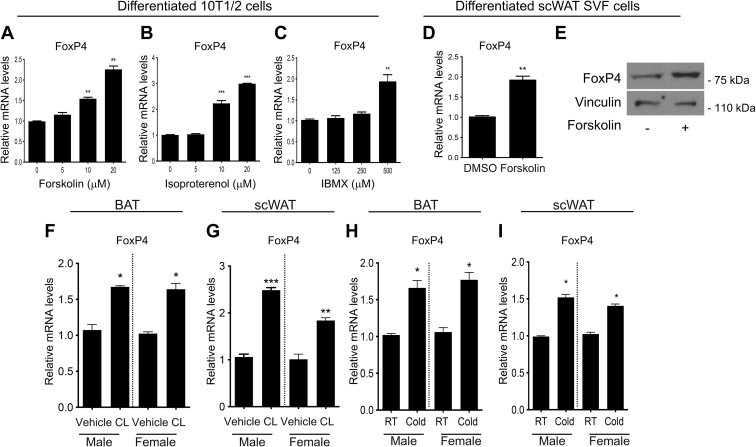
FoxP4 levels are induced by thermogenic stimuli. A–C: Relative FoxP4 mRNA levels in differentiated 10T/2 cells treated for 4 h with different concentrations of forskolin (A), isoproterenol (B), and IBMX indicated in the graph. C: Relative FoxP4 mRNA (D) and protein levels (E) in differentiated scWAT SVF cells treated with 10 μM forskolin. F–I: Relative FoxP4 mRNA levels in BAT and scWAT of 9-week-old male and female mice (n = 4) treated with CL316, 243, or vehicle, for 3 h (F and G) or exposed to cold or room temperature for 6 h (H and I). Results are expressed as mean ± SEM from three independent experiments. ∗*P* < 0.05; ∗∗*P* < 0.005; and ∗∗∗*P* < 0.001. BAT, brown adipose tissue; FoxP4, Forkhead box P4; IBMX, isobutylmethylxanthine; scWAT, subcutaneous white adipose tissue; SVF, stromal vascular fraction.

Given that transcription factors and cofactors such as PGC1α, ZFP516, PRDM16, PPARα, ZNF638 (29, 30), and HSF1 (27, 31) are upregulated in vitro by agents that increase cAMP levels, we tested whether the expression of FoxP4 is induced in vitro in response to pharmacological stimuli. Dose response studies in brown-like differentiated 10T1/2 cells revealed that treatment for 4 h with different concentrations of either forskolin, isoproterenol, or IBMX lead to an increase in FoxP4 mRNA levels in comparison to vehicle-treated cells (Fig. 2A–C). Further analysis performed in brown-differentiated mouse SVF cells confirmed that FoxP4 levels are induced by forskolin treatment (Fig. 2D and E). To assess whether the induction of FoxP4 by thermogenic cues observed in vitro occurs also in vivo, we analyzed the effects of treatment of mice with the β-adrenergic agonist CL316,243 (CL) on FoxP4 levels. As shown in Fig. 2F and G, FoxP4 mRNA is induced in BAT and scWAT in response to 4 h of CL treatment, in parallel to the induction of classic markers of thermogenic function, UCP1 and Cidea (supplemental Fig. S2). To determine whether the levels of FoxP4 are also modulated by exposure to low temperatures, we analyzed fat depots of mice exposed to either room temperature or 4°C for 6 h and demonstrated that FoxP4 is induced by cold in BAT and scWAT of both males and females (Fig. 2H and I). These data suggest that FoxP4 levels are controlled by thermogenic stimuli, both in isolated cells in vitro and in tissues in vivo.

### FoxP4 levels are regulated by HSF1

**Fig. 3:**
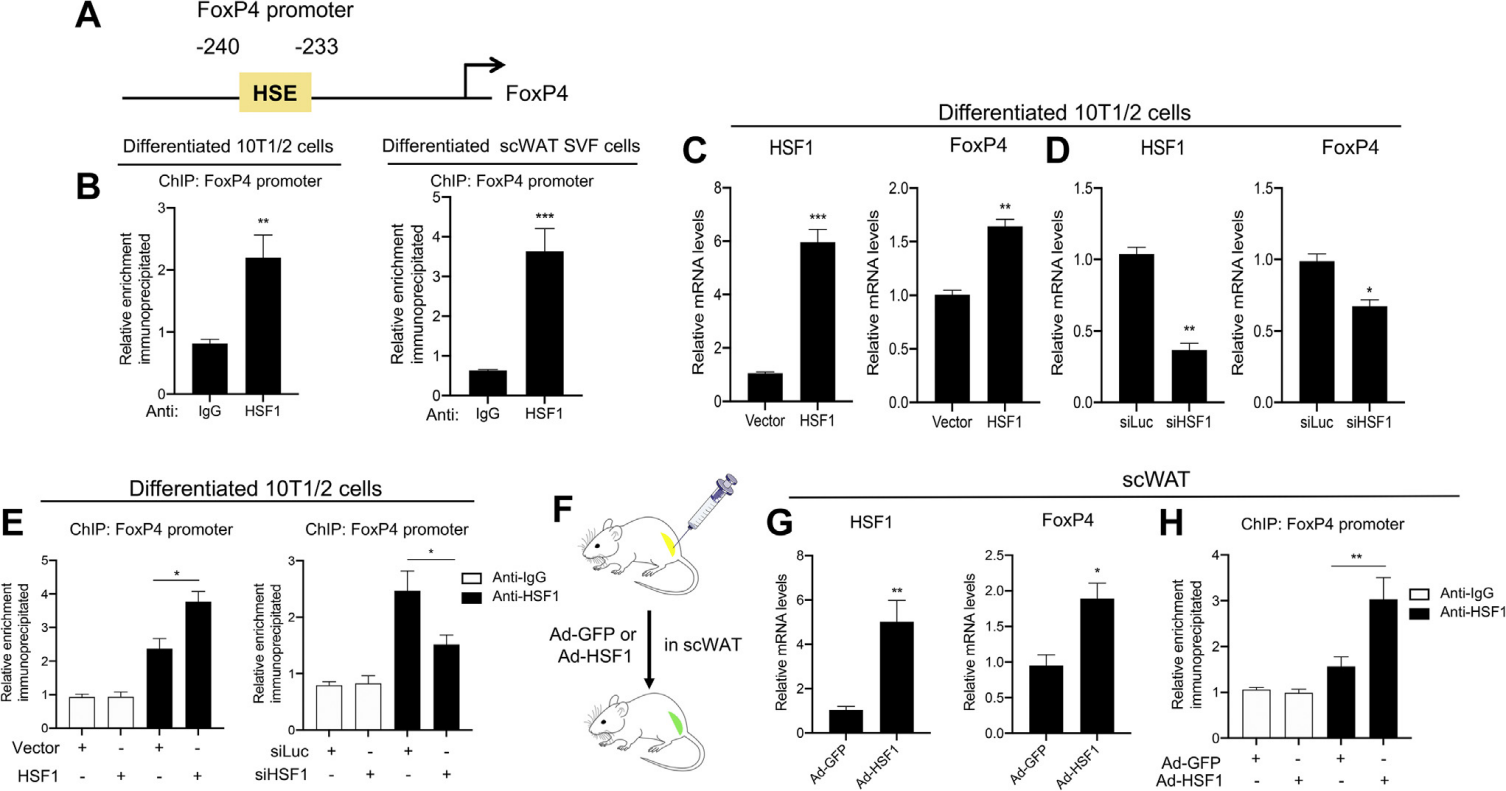
FoxP4 levels are controlled by HSF1. A: In silico analysis using the Genomatix program identified a putative HSE in position −240 to −233 from the FoxP4 transcriptional start site. B: ChIP assays performed in brown-like differentiated 10T1/2 and SVF cells obtained from scWAT. Anti-IgG and anti-HSF1 antibodies were used to assess the occupancy of HSF1 at the putative HSE. C and D: Relative RNA levels of FoxP4 and HSF1 in 10T1/2 differentiated cells with either overexpression (C) or knockdown of HSF1 (D). E: ChIP assays performed in differentiated 10T1/2 cells with either overexpression or knockdown of HSF1. Anti-IgG and anti-HSF1 antibodies were used to assess the occupancy of HSF1 at the putative HSE identified in the FoxP4 promoter. F: Schematic representation of the experimental design. G: mRNA and protein levels of HSF1 and FoxP4 in scWAT of mice injected with either vector or HSF1 adenoviruses (Ad-GFP or Ad-HSF1) (n = 4). H: ChIP assays in scWAT of mice injected with either HSF1 adenovirus (Ad-HSF1) or control (Ad-GFP). Anti-IgG and anti-HSF1 antibodies were used to assess the occupancy of HSF1 at the putative HSE in the FoxP4 promoter. Results are expressed as a mean ± SEM from three independent experiments. ∗*P* < 0.05; ∗∗*P* < 0.005; and ∗∗∗*P* < 0.001. ChIP, chromatin immunoprecipitation; FoxP4, Forkhead box P4; HSE, heat shock element; HSF1, heat shock factor protein 1; scWAT, subcutaneous white adipose tissue; SVF, stromal vascular fraction.

**Fig. 4:**
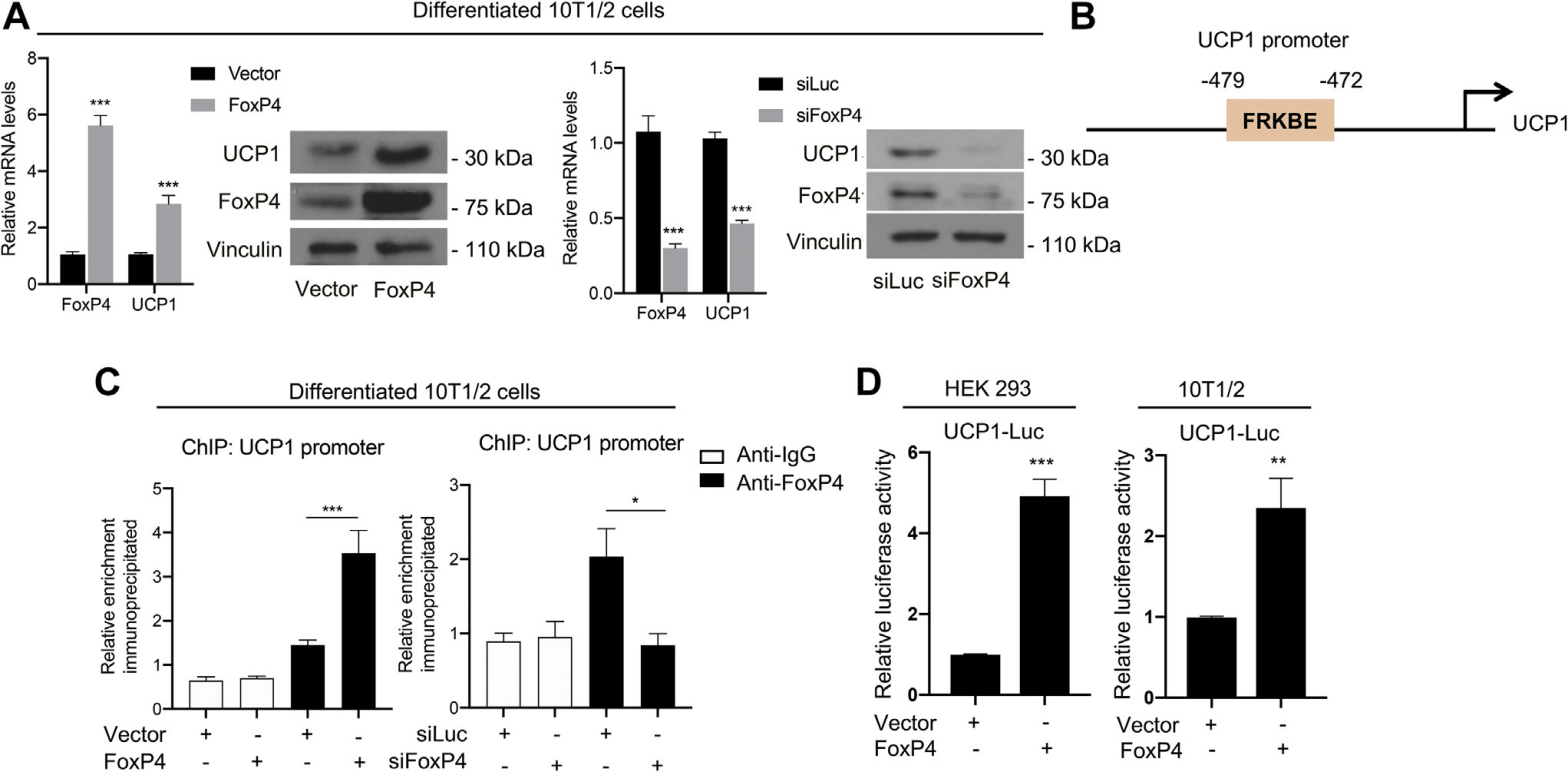
FoxP4 directly controls UCP1 gene expression. A: FoxP4 and UCP1 mRNA and protein levels in 10T1/2 differentiated cells with either FoxP4 overexpression or knockdown. B: In silico analysis using the Genomatix program identified a putative Forkhead-binding motif at -479 to -472 from the UCP1 transcription start site. C: ChIP assays performed in 10T1/2 differentiated cells with either FoxP4 overexpression or knockdown. Anti-IgG and anti-FoxP4 antibodies were used to assess the occupancy of FoxP4 at the putative Forkhead response element identified in the UCP1 promoter. D: Luciferase assay in HEK-293 and differentiated 10T1/2 cells cotransfected with either vector or FoxP4, in the presence of the UCP1 luciferase reporter construct. Results show a representative experiment performed in triplicate. Results are expressed as a mean ± SEM from three independent experiments. ∗*P* < 0.05; ∗∗*P* < 0.005; and ∗∗∗*P* < 0.001. FoxP4, Forkhead box P4; HEK-293, human embryonic kidney 293 cells; UCP1, uncoupling protein 1.

We have previously shown that HSF1 is induced by cold exposure and agents that increase cAMP levels (31). Furthermore, our studies have demonstrated that HSF1 plays a key role in the regulation of thermogenic tissues via induction of the expression of the metabolic coactivator PGC1α. To assess the mechanisms of FoxP4 regulation in adipose tissues, we took a candidate approach and hypothesized that HSF1 may directly control the levels of expression of FoxP4. Our initial in silico screen of the 2 Kb sequence upstream of the FoxP4 transcription start site for putative HSE-binding motifs revealed the presence of one possible HSF1 response element located most proximally, in position −240 to −233 (Fig. 3A), to the transcriptional start site. To further determine whether HSF1 could occupy the FoxP4 promoter at the identified putative HSE, we performed ChIP assays in brown-differentiated 10T1/2 and SVF cells obtained from scWAT. Our results demonstrated that endogenous HSF1 is present at this putative response element (Fig. 3B), but not at the promoter of the β-actin gene, used as a negative control (supplemental Fig. S3). To assess whether HSF1 is sufficient and necessary to induce the expression of FoxP4, we performed gain-of-function and loss-of-function experiments in differentiated 10T1/2 cells. Specifically, overexpression of HSF1 led to the induction of FoxP4 (Fig. 3C), whereas its knockdown was associated with decreased FoxP4 levels (Fig. 3D). ChIP assays performed in differentiated 10T1/2 cells with either overexpression or downregulation of HSF1 confirmed that modulation of HSF1 levels alter HSF1 occupancy at the HSE identified in position −240 to −233 (Fig. 3E). To assess whether ectopic expression of HSF1 leads to the induction of FoxP4 levels also in vivo, scWAT of 9-week-old mice that received bilateral injections twice a week for 2 weeks with either control (Ad-GFP) or HSF1 adenovirus (Ad-HSF1) into scWAT were analyzed for the levels of FoxP4 expression and promoter occupancy. As shown in Fig. 4F–H, we observed an elevation in FoxP4 mRNA levels in the scWAT of mice injected with HSF1, compared with control injected, which was accompanied by an increase in HSF1 occupancy at the endogenous FoxP4 promoter. Altogether, these data demonstrate that HSF1 is both sufficient and necessary for the regulation of FoxP4 levels, both in vitro and in vivo.

### FoxP4 directly controls UCP1 levels

Given our demonstration that FoxP4 levels are regulated in adipose tissues, we assessed the potential relevance of FoxP4 in inducing the expression of the critical marker of brown fat function UCP1. FoxP4 gain-of-function and loss-of-function experiments in differentiated 10T1/2 cells demonstrated that modulation of FoxP4 affects UCP1 mRNA and protein levels, as shown in Fig. 4A but does not affect the early stages of brown fat differentiation (supplemental Fig. S4). To assess whether FoxP4 may be directly involved in the control of the UCP1 promoter, we performed an in silico analysis and identified the presence of a forkhead DNA-binding motif in position -479 to -472 from the UCP1 transcriptional start site (Fig. 4B). To assess whether FoxP4 binds to this response element to directly regulate the expression of UCP1, we performed ChIP assays in differentiated 10T1/2 cells. As shown in Fig. 4C, endogenous FoxP4 binds to the response element present in position -479 to -472, but not at the promoter of β-actin used as a negative control (supplemental Fig. S5). Furthermore, luciferase assays performed in differentiated 10T1/2 and HEK-293 cells demonstrated that FoxP4 regulates the UCP1 promoter (Fig. 4D). Altogether, our data provide the first evidence that FoxP4 directly controls the expression of UCP1.

### FoxP4 regulates thermogenic gene expression and function

**Fig. 5:**
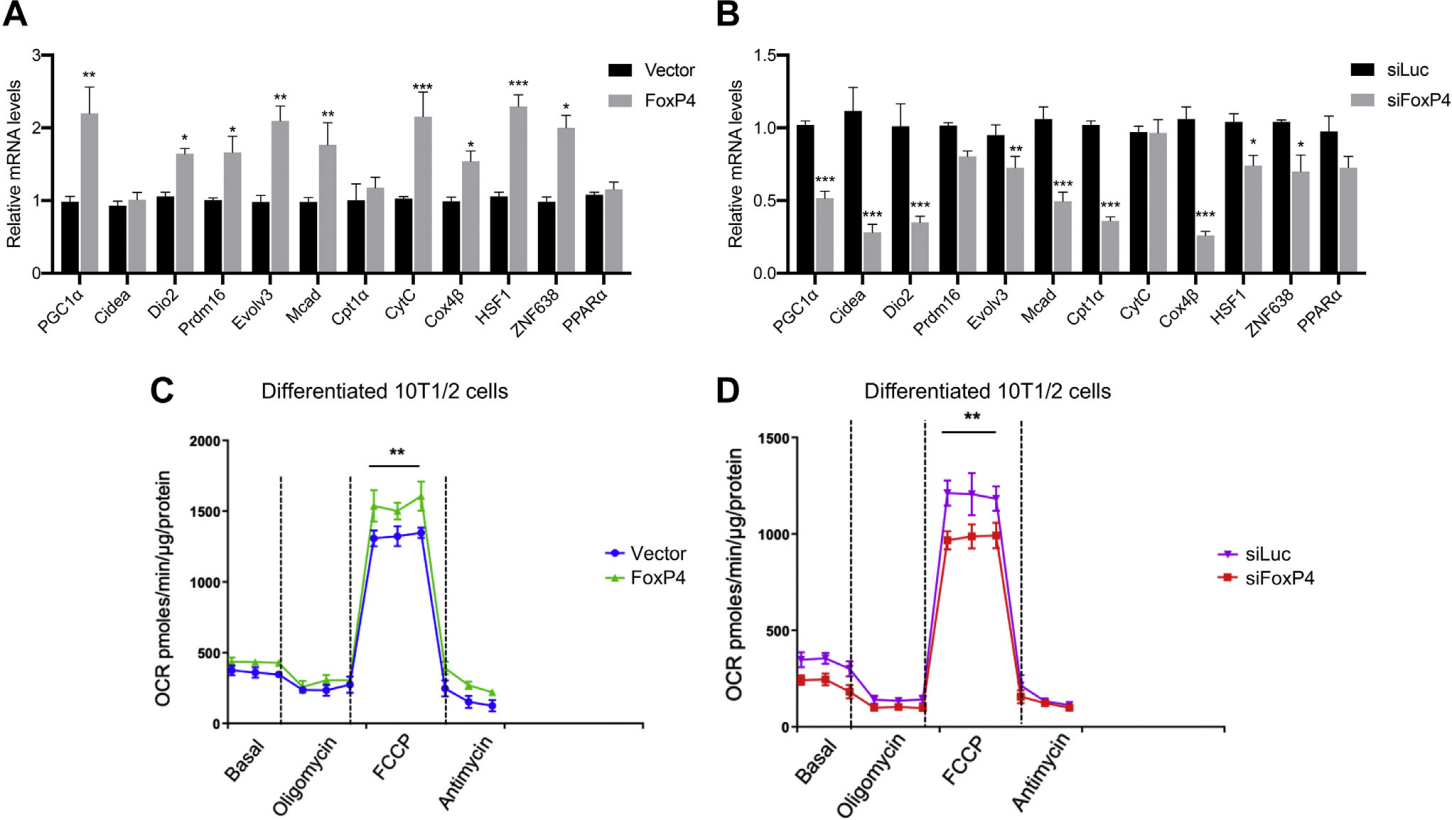
FoxP4 modulates brown fat gene programs and thermogenic function in adipocytes. A and B: Relative mRNA levels in 10T1/2 differentiated cells with either HSF1 overexpression (A) or knockdown of HSF1 (B). Seahorse measurements of oxygen consumption in differentiated 10T1/2 cells with either FoxP4 overexpression (C) or knockdown (D). Results are expressed as a mean ± SEM from three independent experiments. ∗*P* < 0.05; ∗∗*P* < 0.005; and ∗∗∗*P* < 0.001. FoxP4, Forkhead box P4; HSF1, heat shock factor protein 1.

Since FoxP4 regulates the classic thermogenic marker UCP1, we determined the importance of FoxP4 in adipocyte function by assessing the impact of FoxP4 gain-of-function and loss-of-function studies in differentiated 10T1/2 cells on a panel of thermogenic genes. Our gene expression analysis in cells overexpressing FoxP4 revealed that the levels of genes such as PGC1α, Dio2, HSF1, ZNF638, CytC, Cox4β, Prdm16, Evolv3, and Mcad are significantly induced (Fig. 5A). Conversely, downregulation of FoxP4 expression in 10T1/2 differentiated cells via siRNA led to a decrease in the levels of the majority of these markers (Fig. 5B). Given the direct binding observed of FoxP4 to the UCP1 promoter and Foxp4 modulation of the expression of classic markers of thermogenic function, we assessed whether FoxP4-binding motifs may lie upstream of the transcriptional start site of genes such as Cidea and Dio2 by performing Genomatix and Promo analyses. Our studies revealed the presence of putative Forkhead-binding motifs in the promoter regions of these genes (supplemental Table S1), suggesting the possibility that FoxP4 may directly regulate the expression also of Cidea and Dio2. To further determine the impact of FoxP4 on thermogenic fat functionality, we measured oxygen consumption via Seahorse in differentiated 10T1/2 cells with either gain-of-function or loss-of-function studies of FoxP4. As shown in Fig. 5C and D, 10T1/2 cells overexpressing FoxP4 showed higher oxygen consumption compared with controls, whereas knockdown of FoxP4 was associated with reduced levels. These data strongly support the idea that FoxP4 regulates thermogenic fat functionality.

## Discussion

Forkhead box transcription factors have been shown to regulate development and differentiation processes in a number of cell types; however, less is known about the role that these proteins play in adipocyte differentiation and thermogenic functionality. While to date, members of the Foxo, Foxa, and Foxc subfamilies, such as Foxo1 (11, 12, 14, 32), Foxa1 (33), Foxa3 (16, 34), and Foxc2 (32), have been shown to be involved in certain aspects of adipose tissue biology, there is currently very little information on the levels of expression and on the possible role of the members of the FoxP family in adipose tissues. In this report, we show for the first time that FoxP4 is expressed in select adipose depots, that its levels are induced by modulators of cAMP and by cold exposure, that FoxP4 is directly controlled by the heat shock transcription factor HSF1 and that FoxP4 is involved in the regulation of thermogenic gene expression and function.

Our studies demonstrating that FoxP4 is present in adipose tissues is novel given that FoxP4 has been mainly reported to be expressed in neuronal, pulmonary, gut, and T cells (21, 28, 35, 36). More specifically, our data provide new evidence that the basal levels of FoxP4 are selectively enhanced in subcutaneous fat tissue, suggesting a potential distinct role of FoxP4 specifically in this depot. Future gain-of-function and loss-of-function studies of FoxP4 selectively in scWAT may reveal the specific relevance of FoxP4 in the functionality of this adipose depot.

Our data analyzing the inducibility of FoxP4 in response to classic thermogenic cues and promoter occupancy studies in combination with gain-of-function and loss-of-function analyses mechanistically demonstrate that FoxP4 expression is regulated through a functional HSE motif present in its proximal promoter region, both in vitro and in vivo, and that this regulation involves the stress-induced transcription factor HSF1. These data not only specify a novel mechanism of FoxP4 regulation previously not characterized but also expand our current knowledge on the mechanisms through which HSF1 regulates brown fat programs, beyond the role that our laboratory has described for HSF1 as a regulator of PGC1α expression (37). In addition, our studies demonstrate that HSF1 activates thermogenic programs not only via the induction of a coactivation function mediated by PGC1α but also through direct control of the levels of transcriptional regulators impacting on adipose tissue functionality, such as in the case of FoxP4 that we have reported here. Based on our demonstration of the existence of a novel HSF1-FoxP4 axis, it can be hypothesized that the activation of thermogenic fat programs by HSF1 could be mediated by transcription factors directly activated by HSF1, in addition to FoxP4. Future systematic analyses via a combination of RNA-Seq and ChiP-Seq studies in adipose tissues will reveal whether HSF1 controls thermogenic function through select transcription factors in distinct depots.

Our results demonstrate for the first time that FoxP4 is involved in thermogenic functionality via the direct control of the UCP1 promoter through a forkhead-binding motif not previously shown to be involved in the regulation of UCP1 expression. Future studies will determine whether forkhead box factors may complement each other by acting promiscuously at the forkhead-binding motif we have identified at the UCP1 promoter or whether they may compete for their binding in virtue of their differential affinity to this site.

Our data obtained through RNA analysis and Seahorse studies demonstrate that modulation of FoxP4 levels not only affects thermogenic gene expression but also functionally alters oxygen consumption in adipocytes, thereby implicating FoxP4 for the first time in thermogenic fat activity. In conclusion, our studies provide the first demonstration of FoxP4 involvement in adipocyte tissue functionality, neither previously known nor appreciated.

## Data availability

The data underlying this article are available in the article. Additional data underlying this article will be shared on reasonable request to the corresponding author.

## Supplemental data

This article contains supplemental data.

## Conflict of interest

The authors declare that they have no conflicts of interest with the contents of this article.
