# Supplementary material for: The forkhead box transcription factor FoxP4 regulates thermogenic programs in adipocytes
Source: J Lipid Res. 2021 Aug 9;62:100102. doi: 10.1016/j.jlr.2021.100102 (PMC8411233; doi:10.1016/j.jlr.2021.100102)
Supplement: Figures S1–S5 and Table S1 [file mmc1.pdf]

SUPPLEMENTAL INFORMATION

The Forkhead Box Transcription Factor FoxP4 Regulates Thermogenic Programs in Adipocytes

Luce Perie[1], Narendra Verma[1] & Elisabetta Mueller*

Division of Endocrinology, Diabetes and Metabolism, Department of Medicine, New York University Grossman School of Medicine, New Science Building, Room 612, 435 E 30th Street, New York, NY, 10016, USA

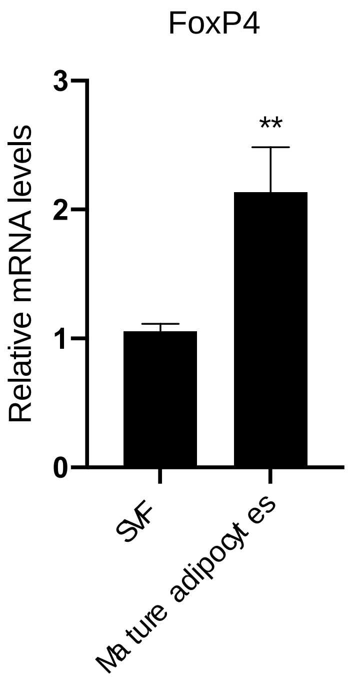

Figure S1. FoxP4 levels are higher in mature adipocytes than in SVF cells.

Relative FoxP4 mRNA levels in mature adipocytes and SVF cells obtained from scWAT of 9 weeks-old male mice. Results are expressed as a mean ± SEM from three independent experiments and ** p value < 0.005.

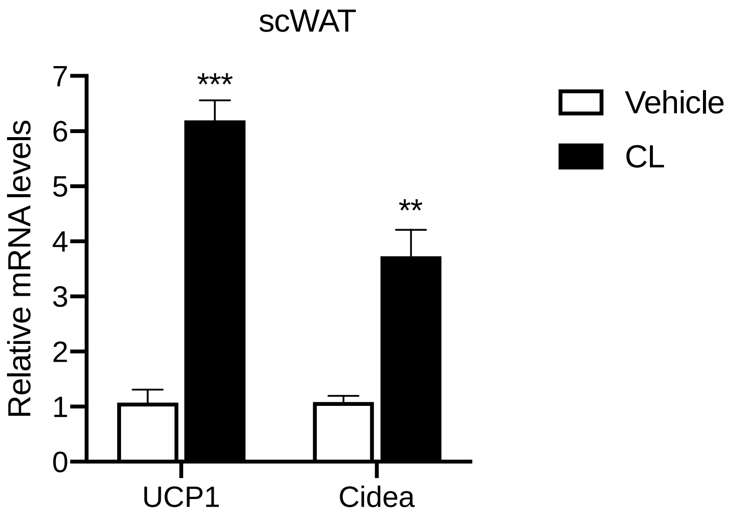

Figure S2. Induction of brown fat markers in scWAT of mice treated with CL.

Relative UCP1 and Cidea mRNA levels in scWAT of 9-week-old male treated with vehicle or CL316, 243 for 3 hours. Results are expressed as a mean ± SEM from three independent experiments and * *p* value < 0.05; ** *p* value < 0.005; *** *p* value < 0.001.

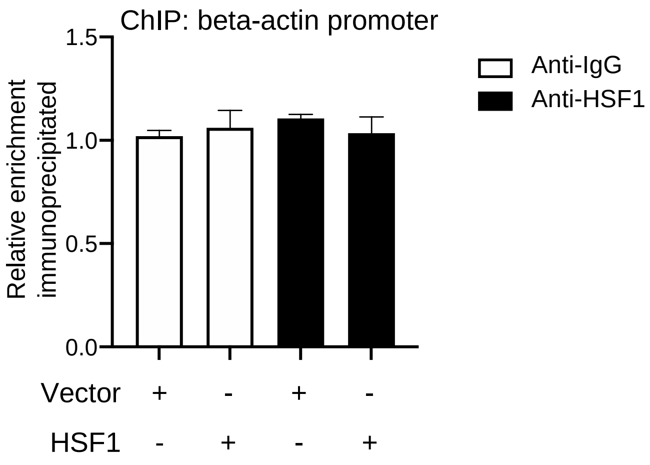

Figure S3. ChIP assay at the promoter of the β-actin gene used as a control in differentiated cells ectopically expressing either vector or HSF1.

ChIP assay performed in brown like differentiated 10T1/2 with either an anti-IgG or an anti-HSF1 antibody at the β-actin promoter, used as a negative control. Results are expressed as a mean ± SEM from three independent experiments.

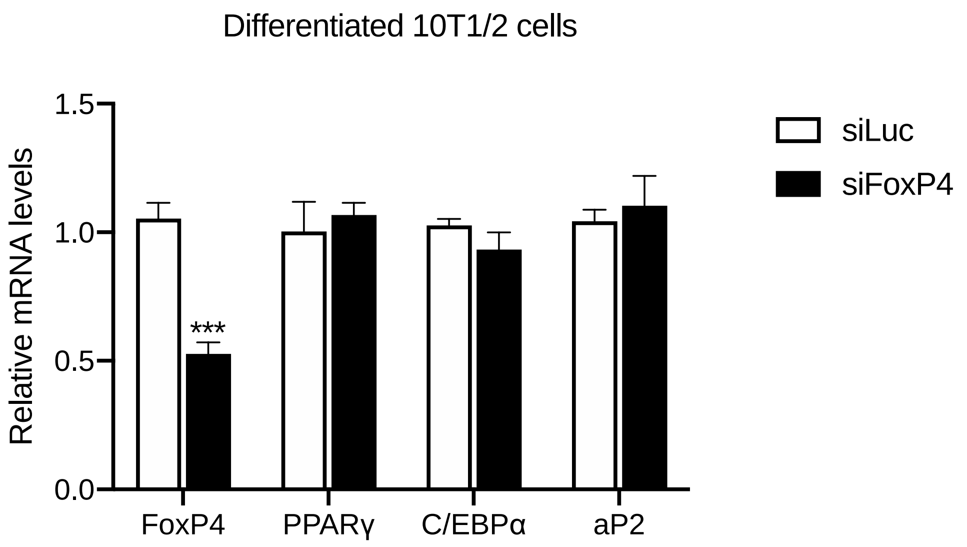

Figure S4. Down-regulation of FoxP4 levels does not affect brown adipogenesis.

mRNA levels of FoxP4, PPARγ, CEBPα and aP2 in brown differentiated 10T1/2 cells transfected with either siLuc or siFoxP4, 24 hours prior to the induction of differentiation. Results are expressed as a mean ± SEM from three independent experiments and * p value < 0.05; ** p value < 0.005; *** p value < 0.001.

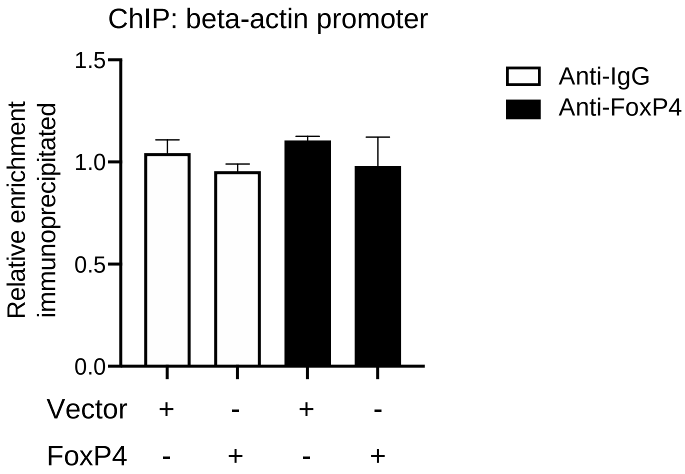

Figure S5. ChIP assay at the promoter of the β-actin gene used as a negative control in differentiated cells ectopically expressing either vector or FoxP4.

ChIP assay performed in brown like differentiated 10T1/2 with either an anti-IgG or an anti-FoxP4 antibody at the β-actin promoter, used as a negative control. Results are expressed as a mean ± SEM from three independent experiments.

Table S1. ***In silico*** **analysis identifies putative Forkhead binding motifs within 2Kb of the region upstream of the transcription start site of Cidea and Dio2.**

Gene promoter (2kb)	Transcription factor	Binding motifs	Location
Cidea	Foxp3	AGACATTTT	1154 - 1162
Cidea	Foxp3	TCCCATTTT	1188-1196
Cidea	Foxp3	TCCAATTTT	1533-1541
Cidea	Foxa4a	TGTTTAAATGA	991-1001
Dio2	Foxp3	AAAATGTCA	226 - 234
Dio2	Foxp3	TAGTATTTT	636-644
Dio2	Foxp3	CTTTATTTT	975-986
Dio2	Foxp3	AAAATGCAT	1894-1902
Dio2	Foxa4a	TGTTTAAATAA	316-326
Dio2	Foxa4a	CTCATAAATAA	581-591
